# Cecal duplication cyst presenting with acute abdomen: a case report

**DOI:** 10.11604/pamj.2022.41.280.33731

**Published:** 2022-04-07

**Authors:** Menawar Dajenah, Faisal Ahmed, Abdullatif Almohtadi, Anessa Thabet, Khaled Ghaleb, Fayed Al-Yousofy, Fawaz Mohammed

**Affiliations:** 1 Department of General Surgery, School of Medicine, Ibb University of Medical Science, Ibb, Yemen,; 2 Urology Research Center, Al-Thora General Hospital, Department of Urology, School of Medicine, Ibb University of Medical Science, Ibb, Yemen,; 3 Department of Radiology, School of Medicine, Ibb University of Medical Science, Ibb, Yemen,; 4 Department of Gynecology, School of Medicine, Ibb University of Medical Science, Ibb, Yemen,; 5 Department of Internal Medicine, School of Medicine, Ibb University of Medical Science, Ibb, Yemen,; 6 Department of Pathology, Faculty of Medicine, Taiz University of Medical Science, Taiz, Yemen,; 7 Department of Orthopedy, School of Medicine, Ibb University of Medical Science, Ibb, Yemen

**Keywords:** Cecal duplication cyst, intestinal obstruction, adults, case report

## Abstract

Cecal duplication cyst is a rare congenital malformation with a few reported adult cases. We present a 23-year-old man who presented with low-grade fever, constipation, and right lower quadrant pain for three days. An abdominal computed tomography scan showed a cystic mass of 8.8x7.5x6 cm adjoining to the posterior wall of the cecum. The patient underwent abdominal laparotomy, and a right hemicolectomy was performed with resection of the duplication cyst. The histopathological study confirmed the diagnosis of a non-communicating cecal duplication cyst. In conclusion, it is essential to include cecal duplication cyst in the differential diagnosis of the acute abdomen to guarantee the best treatment strategy.

## Introduction

Cecal duplication cysts are the rarest gastrointestinal duplication cysts [1]. The vast majority of cases were discovered during childhood and before the age of two years, with only a few cases mentioned in adults [2]. Cecal duplication cyst presents with various clinical symptoms, including abdominal mass, intestinal obstruction, rectal hemorrhage, acute appendicitis, and intestinal obstruction. However, most cases (50%) present with a palpable mass [2,3]. Most duplication cysts communicate with the gastrointestinal tract, with limited published cases of non-communicating duplication cysts [4]. We present a 23-year-old man who presented with abdominal pain and was diagnosed with cecal duplication cyst. The adult age at symptom onset, non-communicating cyst, and cecal location contribute to the rarity of this case.

## Patient and observation

**Patient information:** a 23-year-old male presented to the Emergency Department of Al-Manar Hospital, Ibb, Yemen, in September 2021 with constipation, low-grade fever, and abdominal pain in the last three days. The pain was located in the right lower hypochondrium region-no history of vomiting, hematuria, or dysuria. There is no medical history of an inherited or chronic disease. The patient mentioned repeated hospital admission due to abdominal pain and constipation in the last two years and took medication without improvement.

**Clinical findings:** on physical examination, the pulse rate: 60 beats per minute, the respiratory rate: 24 per min, and the oral temperature: 37.8°C. Abdominal examination revealed moderate abdominal distention, mild tenderness in the right lower hypochondrium region, and the bowel movements were normal in auscultation.

**Figure 1:**
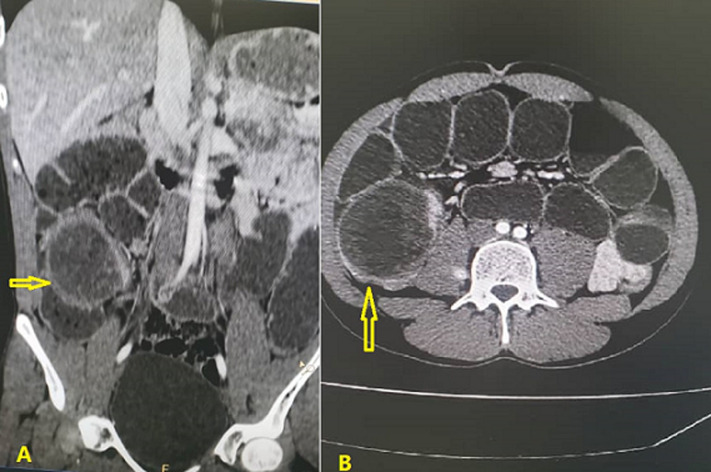
A) coronal CT scan showing the cecal duplication cyst (arrow); B) axial CT shows a non-communicating cecal duplication cyst measuring 8.8x7.5x6 cm (arrow) and dilated small bowel

**Diagnostic assessment:** blood tests revealed a total white blood cell count of 12x10^3^/ml with moderate leukocytosis, hemoglobin: 14 g/dl; C-reactive protein: 13.9 mg/dl, echinococcus antibody: negative, all other blood investigations were within the normal range. Abdominal ultrasonography (US) showed an ovoid cystic mass lesion measuring 9.5x8 cm within fluid content in the right lower hypochondrium region, adjacent to the cecum. The intravenous computerized abdominal tomography scan showed a well-defined, thick hypodense cystic lesion that obstructed the lumen at the level of the cecum measured 8.8x7.5x6 cm, suggestive for giant cystic lesion likely duplication cyst or hydatid cyst. There was moderate dilatation in the small bowel loops measured 5 cm in diameter proximal to the mentioned cystic lesion. However, the bowel loops appeared with normal wall enhancement (Figure 1).

**Figure 2:**
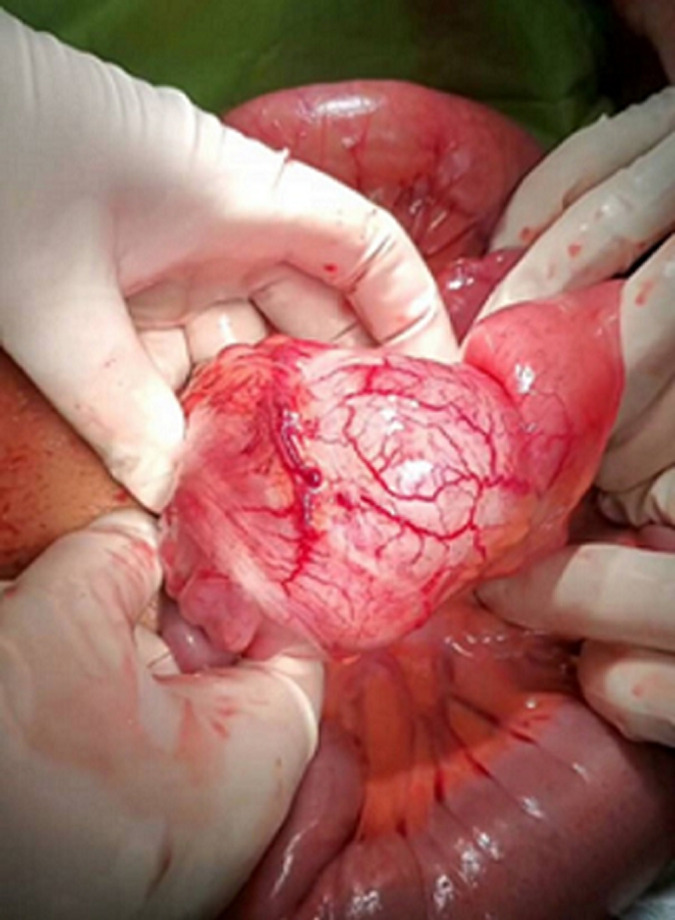
intraoperative photo showing the non-communicating cecal duplication cyst

**Therapeutic interventions:** the patient was admitted for emergency surgical exploration. After general anesthesia, the abdomen was opened via an open midline incision; a cystic mass close to the cecum originating from its mesenteric border was identified. The exterior cyst surface was reddish-brown (Figure 2). The cystic resection and right hemicolectomy were done. Then, the drain was inserted into the abdominal cavity and the fascia and abdominal wall were closed.

**Figure 3:**
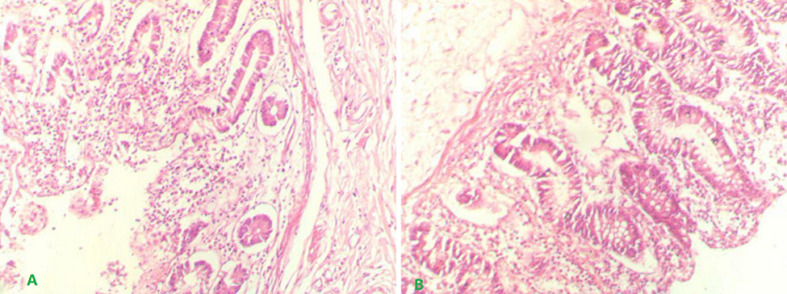
histopathologic investigation shows two lumens with mucosa and submucosa shared by the muscular layer-the duplication gut (A) and the original lumen (B)

**Follow-up and outcome:** the patient was discharged from the hospital on the fifth postoperative day after an unremarkable postoperative period. An enteric cyst and reactive lymphoid hyperplasia in 15 lymph nodes were discovered during the histopathological examination. On microscopic examination, the cyst wall was unrelated to the intestinal lumen, and intestinal mucosa and muscularis propria were found, which were consistent with the cecal duplication cyst (Figure 3). Within three months of follow-up, the patient remained symptom-free.

**Patient perspective:** the patient was happy with the successful outcome of the surgery.

**Informed consent:** a written informed consent was obtained from the patient for participation in our study.

## Discussion

Duplication cysts are uncommon hereditary anomalies that can arise throughout the gastrointestinal tract. Although its etiology is unknown, numerous theories have been proposed. The most widely accepted hypotheses are fetal intestinal diverticulum persistence, a defect in primordial intestine recanalization, incomplete copulating, and notochord detachment [5]. The most common site of duplication cyst is the ileum, followed by the esophagus, and the jejunum. However, only 6.8% of colorectal duplication cysts have been reported [6]. In another study, the incidence rate of duplication cysts in the gastrointestinal tract was 31.5% located in the ileum, 30.2% found in the ileocecal valve, 9.6% located in the duodenal part, 8.2% located in the stomach, 8.2% located in the jejunum, and only one of 73 patients had a duplication cyst in the cecum [7].

Additionally, the majority of duplication cysts are communicating with the normal intestinal wall, with only very few cases of enteric non-communicating duplication cysts have been reported [4]. It is most commonly seen in females and over 80% of duplication cysts present in childhood before two years and a small percentage of people may be asymptomatic until adulthood [4]. Our patient was an adult male and had a cecal non-communicating duplication cyst. Depending on their location, type, and diameter, duplication cysts can be asymptomatic or cause an acute abdomen. They are frequently characterized by vomiting, abdominal distention, and palpable abdominal mass-however, acute abdominal manifestations such as intussusception, perforation, intestinal obstruction, and volvulus are rare. Additionally, most duplication cysts that cause acute abdomen are colonic [8]. Our patient suffered from abdominal distension and constipation, resulting from advanced intestinal obstruction due to cecal duplication cyst.

The radiologic modalities for diagnosing the duplication cysts are the US, Computed Tomography (CT) scan, Magnetic resonance imaging (MRI), and Technetium pertechnetate scan. The US with double-wall sign had more than 95% specificity and an 85-100% positive predictive value. However, it is operator-dependent and may be difficult to distinguish from intussusception [9]. Computed tomography scan is the most effective modality for detecting duplication cysts. The details of duplication cyst on CT are typically double-walled, with an internal hyperechoic layer reflecting the mucosa and submucosa and an external hypoechoic layer with rounded edges linked to the muscularis propria as reported in our case [10]. The differential diagnoses of duplication cyst comprise acute appendicitis, intussusception, intestinal cyst, choledochal cyst, and lymphoma [2].

Segmental surgical removal and end-to-end anastomosis are preferred for small duplication cysts. Suppose the lesion is extensive and tubular and cannot be excised to preserve bowel length. In that case, other procedures such as enucleation, marsupialization, excision of the mucosal layer may be used [1,4,11]. Some authors advocate removal to avoid future complications, such as malignant transformation [12]. Attachment to the gastrointestinal tract, smooth muscle in the wall, and an epithelial lining approximating some part of the gastrointestinal tract are the histopathological criteria for diagnosing the duplication cyst [5]. With the right hemicolectomy and a complete histopathological investigation of the specimen, we ruled out the possibility of an underlying malignancy, and our patient's outcome was adequate, and no further follow-up was required. Al-Shaibi and associations reported a similar approach and result [2].

## Conclusion

Cecal duplication cysts are rare, with only a limited case reported in adults, and should be considered in the differential diagnosis of acute abdomen. Early surgical resection is the best option because it reduces the risk of future consequences like perforation, hemorrhage, obstruction, and malignant transformations.

## References

[1] Saxena R, Pathak M, Sinha A (2020). Caecal duplication cyst: a rare disease with variable presentation and its management in the era of laparoscopy. J Minim Access Surg.

[2] Al-Shaibi MA, Raniga SB, Asghar ANM, Al Tubi IS (2019). Caecal duplication cyst leading to intussusception in an adult. BMJ Case Rep.

[3] Liu R, Adler DG (2014). Duplication cysts: diagnosis, management, and the role of endoscopic ultrasound. Endosc Ultrasound.

[4] Radhakrishna V, Rijhwani A, Jadhav B (2018). Cecal duplication: a mimicker of intussusception: a case report and review. Ann Med Surg (Lond).

[5] Macpherson RI (1993). Gastrointestinal tract duplications: clinical, pathologic, etiologic, and radiologic considerations. Radiographics.

[6] Mourra N, Chafai N, Bessoud B, Reveri V, Werbrouck A, Tiret E (2010). Colorectal duplication in adults: report of seven cases and review of the literature. J Clin Pathol.

[7] Puligandla PS, Nguyen LT, St-Vil D, Flageole H, Bensoussan AL, Nguyen VH (2003). Gastrointestinal duplications. J Pediatr Surg.

[8] Erginel B, Soysal FG, Ozbey H, Keskin E, Celik A, Karadag A (2017). Enteric duplication cysts in children: a single-institution series with forty patients in twenty-six years. World J Surg.

[9] Verma S, Bawa M, Rao KL, Sodhi KS (2013). Caecal duplication cyst mimicking intussusception. BMJ Case Rep.

[10] Lee NK, Kim S, Jeon TY, Kim HS, Kim DH, Seo HI (2010). Complications of congenital and developmental abnormalities of the gastrointestinal tract in adolescents and adults: evaluation with multimodality imaging. Radiographics.

[11] Oudshoorn JH, Heij HA (1996). Intestinal obstruction caused by duplication of the caecum. Eur J Pediatr.

[12] Blank G, Königsrainer A, Sipos B, Ladurner R (2012). Adenocarcinoma arising in a cystic duplication of the small bowel: case report and review of literature. World J Surg Oncol.

